# Reframing tumor bed evaluation in non-small cell lung cancer: histopathological challenges and future directions in the era of neoadjuvant immunotherapy

**DOI:** 10.3389/fonc.2026.1716992

**Published:** 2026-02-04

**Authors:** Federica Pezzuto, Eleonora Faccioli, Omar El Mnif, Giuseppe Maggioni, Francesca Lunardi, Francesco Fortarezza, Chiara Giraudo, Marco Schiavon, Laura Bonanno, Giulia Pasello, Andrea Dell’Amore, Fiorella Calabrese

**Affiliations:** 1 Department of Cardiac, Thoracic, Vascular Sciences, and Public Health, University of Padova, Padova, Italy; 2 University Hospital of Padova, Padova, Italy; 3 Pathology Unit, Faculty of Medicine of Tunis, University of Tunis El Manar, Tunis, Tunisia; 4 Department of Surgery, Oncology and Gastroenterology, University of Padova, Padova, Italy; 5 Oncologia Medica 2, Institute of Oncology Veneto (IOV), Padova, Italy

**Keywords:** immunotherapy, major pathologic response, non-small cell lung cancer, pathologic complete response, pathologic response, tumor bed

## Abstract

The introduction of neoadjuvant immunotherapy in non-small cell lung cancer (NSCLC) has led to complex tumor responses that challenge conventional pathological assessment. Thus, traditional endpoints such as major pathological response (MPR) and pathological complete response (pCR) may become insufficient to capture the full spectrum of immune-mediated changes. Indeed, these parameters were originally developed in the context of cytotoxic chemotherapy and may not reflect immune-mediated phenomena and stromal remodeling which can significantly alter the appearance of the tumor bed. As a result, MPR and pCR may underrepresent the true extent of biological response in patients treated with immunotherapy. This review outlines current limitations in morphologic evaluation and highlights the need for immune-adapted criteria. Furthermore, it explores the additional value of digital pathology and AI that offer objective and reproducible quantification of histologic features. Integrating these tools with radiologic and molecular data supports a multidimensional approach to response assessment, aiming to refine prognostication, guide adjuvant therapy, and ensure consistency in clinical trial designs.

## 1 Introduction

Non-small cell lung cancer (NSCLC) is one of the most challenging malignancies, accounting for approximately 85% of cancer diagnoses globally (1, 2). Despite significant advances in early detection, refinement of surgical techniques, and continuous development of systemic therapies (1–3), Lung cancer remains the leading cause of cancer mortality worldwide (18.4% of all cancer deaths), and even in resected early-stage NSCLC, recurrence or death occurs in 8–66% of patients (1). The primary barriers to the improvement of patient outcomes include the advanced stage at the time of initial diagnosis, intratumoral and intertumoral heterogeneity (4, 5), and the complex interplay of intrinsic and acquired resistance mechanisms at both cellular and molecular levels (6–8).

NSCLC includes several histological subtypes, primarily adenocarcinoma and squamous cell carcinoma, each with its own morphological, immunophenotypic, and molecular features (9–11). The intrinsic complexity of these tumors significantly contributes to variable responses to therapy. For example, squamous cell carcinomas generally demonstrate higher rates of response after neoadjuvant chemotherapy or chemo-immunotherapy compared with adenocarcinomas, reflecting biological differences in tumor architecture, mutational burden, and immune microenvironment (4, 5, 12) that may not be fully captured traditional morphological evaluations (9, 13).

Recently, the integration of immunotherapy into neoadjuvant treatment protocols has deeply modified the therapeutic approach to NSCLC (1, 13, 14). Immune checkpoint inhibitors (ICIs), specifically those targeting programmed death-1 (PD-1) and its ligand (PD-L1), have changed the management of advanced NSCLC due to their ability to induce durable anti-tumor immune responses (1, 10, 15). This marked clinical efficacy in metastatic disease has motivated considerable interest in exploring their use at earlier stages, particularly in the neoadjuvant setting (2, 13, 16).

The scientific rationale for neoadjuvant immunotherapy is composite, involving immunological, pathological, and clinical considerations. Firstly, immunotherapy aims to enhance surgical outcomes by reducing tumor size, thus converting initially borderline-resectable tumors into completely resectable lesions (2, 13, 16). Moreover, it exploits the intact tumor microenvironment to prime a robust and systemic anti-tumor immune response (5, 6, 17). In the pre-surgical context, the primary tumor serves as a rich reservoir of tumor-specific neoantigens. Immune checkpoint blockade facilitates the activation, expansion, and diversification of T-cell populations within this antigen-rich environment, promoting systemic immunological memory (6–8). This systemic immune activation is particularly beneficial for targeting micrometastatic disease, which may represent a source of disease recurrence post-surgery (1, 15). The integration of these advanced pathological knowledge with the detection of emerging biomarkers and innovative diagnostic tools can significantly enhance the precision and prognostic power of pathological assessments (9, 18, 19).

## 2 Histopathological assessment: definitions, criteria, and challenges

The histopathological assessment of tumor response after neoadjuvant treatment in NSCLC traditionally involves the quantification of residual viable tumor cells within the tumor bed (9–11, 20, 21). Two principal pathological response endpoints are considered as critical surrogate markers for therapeutic efficacy: major pathological response (MPR), defined as 10% or fewer viable tumor cells, and pathological complete response (pCR), characterized by the complete absence of detectable tumor cells at microscopic examination (10, 13, 16). Importantly, some studies have shown that the frequency of achieving MPR differs by histological subtype, with squamous cell carcinoma generally exhibiting higher MPR rates than adenocarcinoma (18, 22). These response measures are now firmly established in clinical practice and trials, owing to their consistent correlation with improved patient survival and lower recurrence rates (2, 15, 16, 23).

The substrate for adopting these pathological criteria originated from experiences primarily involving cytotoxic chemotherapy, which typically induces quantifiable effects, mainly extensive tumor cell death (11, 23). In this context, residual tumor viability provides an intuitive and directly interpretable metric for response (2, 16). Standardized recommendations, including those proposed by the International Association for the Study of Lung Cancer (IASLC), were developed primarily under this paradigm, emphasizing meticulous specimen handling, rigorous gross tumor bed sampling, precise histologic mapping, and quantification of residual neoplastic elements (9, 10, 13). This approach has significantly enhanced the reproducibility of response assessments across institutions, providing consistent diagnostic criteria for pathologists in clinical practice and research settings (9, 15).

While traditional pathological response assessment criteria have provided advancements for evaluating NSCLC treatment efficacy, their limitations have emerged with the widespread adoption of immunotherapy in neoadjuvant settings (10, 13, 15). Specifically, MPR and pCR rely predominantly on quantifying residual viable tumor, a parameter that may not fully cover the immune-mediated regression patterns and stromal alterations that are characteristic of immunotherapy. In addition, immunotherapy induces prominent inflammation, necrosis, and stromal fibrosis that may closely resemble residual viable tumor or conversely obscure small foci of persistent disease, making morphological assessment particularly challenging. Comprehensive identification and accurate interpretation of immunotherapy-induced histopathological alterations require novel, immune-adapted response criteria supported by standardized histologic definitions (5, 9, 17, 24).

## 3 Chemoimmunotherapy: comparative pathological and clinical outcome

Historically, platinum-based chemotherapy regimens have constituted the cornerstone of neoadjuvant therapy for locally advanced NSCLC (1, 2). These regimens primarily act through direct cytotoxic mechanisms, inducing DNA damage in rapidly proliferating neoplastic cells, triggering apoptosis and necrosis (11, 23). Pathologically, chemotherapy-treated tumors typically demonstrate extensive coagulative necrosis, reduced cellularity, and variable degrees of stromal fibrosis (9–11).

Despite the established role of platinum-based chemotherapy, the overall efficacy in achieving significant pathological responses such as MPR or pCR has been relatively modest (2, 16, 25). A comparative evaluation of regimens, specifically etoposide-cisplatin and docetaxel-cisplatin, demonstrated improved local tumor control rates (2). Nevertheless, even these optimized chemotherapy protocols rarely achieved complete tumor eradication, as evidenced by persistently limited absolute rates of MPR and pCR (15, 16, 25).

The integration of immunotherapy, specifically ICIs, into neoadjuvant regimens has fundamentally transformed both clinical and pathological outcomes in NSCLC (13, 15, 16). Immunotherapy primarily acts by modulating host immune responses to effectively recognize and eliminate tumor cells (5–7). Immune checkpoint blockade, enhances antitumor immunity by reactivating exhausted tumor-infiltrating lymphocytes (TILs) and facilitating their expansion and cytotoxic function (8, 17, 26). Immunotherapy has significantly increased the rates of pathological responses in the neoadjuvant setting, significantly surpassing chemotherapy alone (2, 15, 16). Clinical studies demonstrated superior pathological and clinical outcomes with combined chemo-immunotherapy protocols (14, 16). Specifically, these combinations yielded higher MPR and pCR rates, translating into improved overall survival and disease-free survival (1, 13, 25, 27). Additionally, chemo-immunotherapy-treated tumors commonly display extensive apoptotic tumor cell debris, evidence of ongoing immune-mediated cell death. These morphologic patterns reflect not only the tumor destruction but also the improved immune surveillance (9, 11, 23, 28–32).

**Figure 1:**
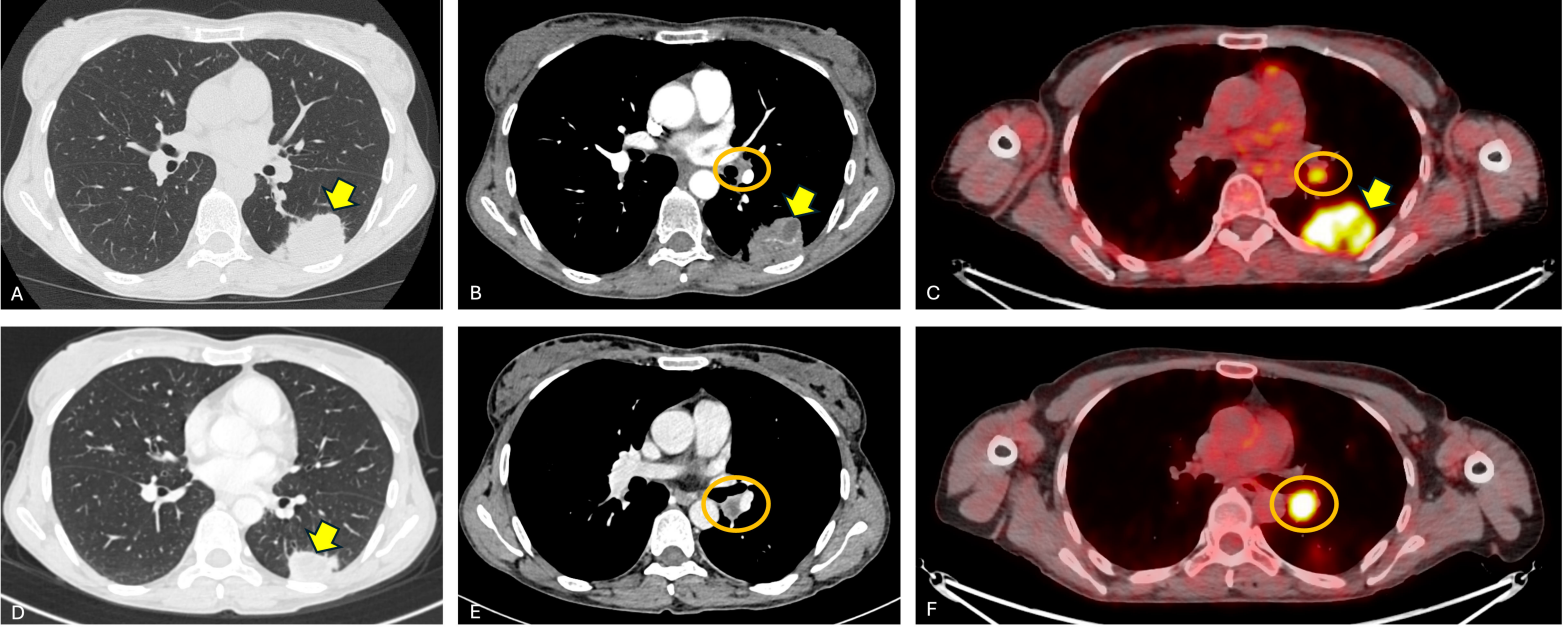
Index case of pseudoprogression after neoadjuvant immunotherapy. A female patient with stage IIIA (cT3N1) non–small cell lung cancer of the left lower lobe underwent four cycles of chemo-immunotherapy before surgical resection. Baseline imaging: **(A)** lung window of the CT scan showing a left lower lobe pulmonary mass (yellow arrow); **(B)** contrast enhanced mediastinal window of the CT scan showing an enlarged left hilar lymph node (orange circle); **(C)** [18F] FDG-FDG/PET CT demonstrating intense uptake in both the primary lesion (SUVmax 15) and the hilar node (SUVmax 4). Post-treatment imaging: **(D)** lung window of the CT scan showing volumetric reduction of the primary tumor (yellow arrow); **(E)** contrast enhanced mediastinal window of the CT scan showing necrotic changes in the left hilar node (orange circle); **(F)** [18F] FDG-PET/CT demonstrating decrease of the FDG uptake of the primary lesion (SUVmax 11) and paradoxical increase in metabolic activity of the hilar node (SUVmax 14.9, orange circle), consistent with pseudoprogression.

Traditional pre-surgical assessments may significantly underestimate efficacy in tumors exhibiting immune engagement but limited viable tumor (9, 10, 13). Conversely, primary tumors and especially hilar-mediastinal lymph nodes may appear radiologically enlarged with increased metabolic activity at [18F] FDG-PET/CT scan due to increased immune infiltrate (**Figure 1**). Pathologically, these tumors often show extensive regression, with high levels of inflammation and fibrosis (6, 8, 26) that maybe better quantified by AI-based pathology tools (5, 17, 25, 28). Clinically, the ability to distinguish pathological responders increases tailored postoperative management. Patients with strong immune-driven responses may avoid overtreatment, while others may benefit from adjuvant strategies or trial enrollment (15, 19).

## 4 Histopathological and molecular predictors of response

**Figure 2:**
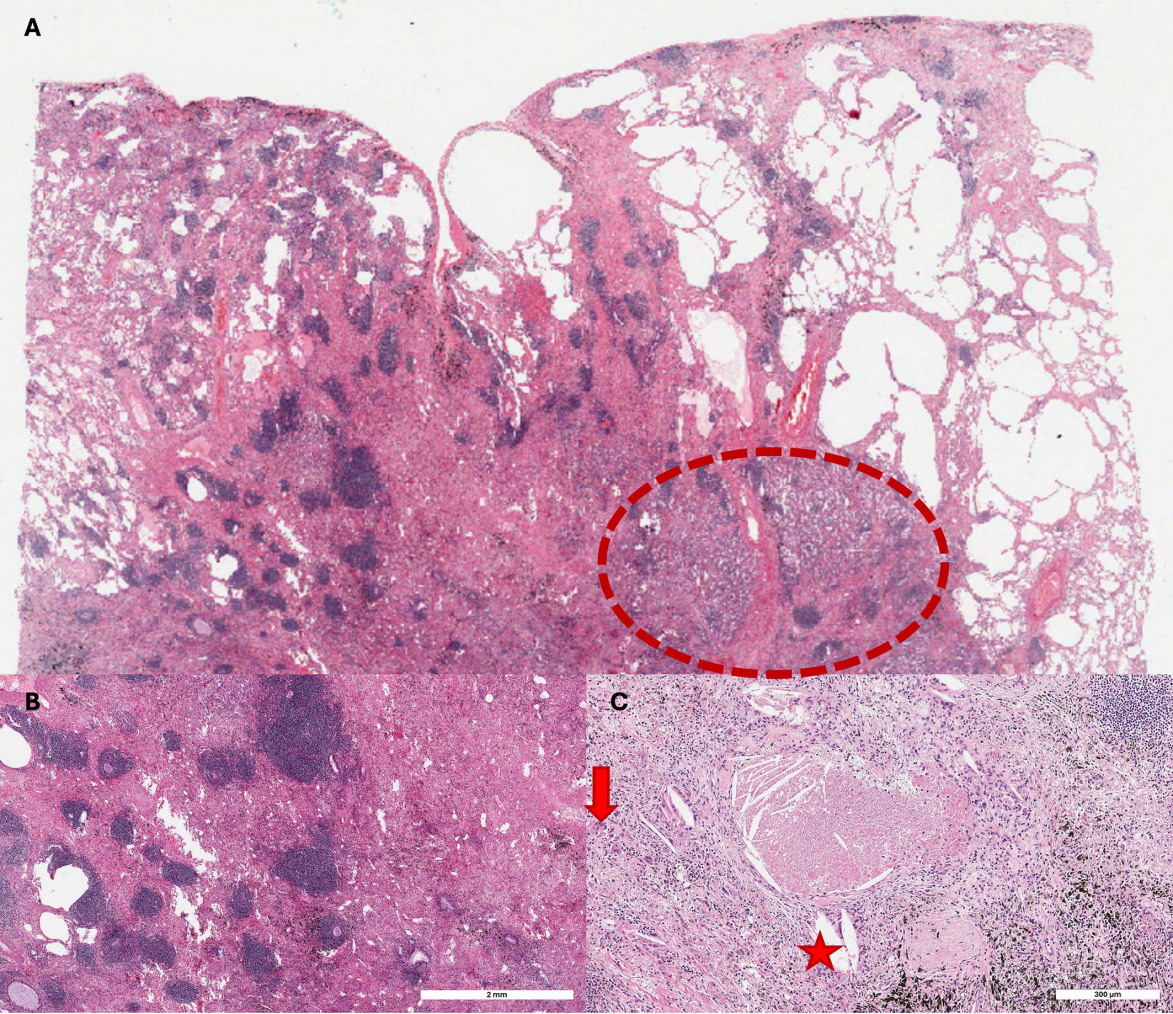
Representative images illustrating the key features of immunotherapy-induced response patterns. [**(A)**, hematoxylin and eosin, 2x original magnification [Panoramic view of the tumor bed (hematoxylin and eosin, 2x original magnification); the dotted circle highlights the small focus of viable tumor cells within a predominantly inflammatory and fibrotic background. Dense lymphocytic aggregates (left side) adjacent to regressing tumor [**(B)**, hematoxylin and eosin, scale bar: 2 mm]; multinucleated giant cells (red arrow) and cholesterol clefts (red star) within fibrotic stroma [**(C)**, hematoxylin and eosin, scale bar: 300 μm].

Immunotherapy induces regression patterns distinct from chemotherapy, which causes direct cytotoxic necrosis. Tumors treated with ICIs show dense lymphocytic infiltrates (CD8+, CD4+, B cells) often organized as tertiary lymphoid structures (TLS). TLS, with germinal centers and T-cell zones, are predictors of effective immune response and favorable outcomes (11, 23, 24). Beyond lymphocytic aggregates and TLS, immunotherapy-treated tumors often show granulomatous inflammation with histiocytes and giant cells, reflecting improved immune activation (6, 9). Foamy macrophages, actively clearing apoptotic tumor cells, and cholesterol clefts, residues of lipid-rich necrosis, are additional indicators of effective immune-mediated regression (5, 11, 23) (**Figure 2**).

Stromal fibrosis, with dense collagen and myofibroblastic proliferation, is frequent after immunotherapy and often indicates regression. However, these immune-mediated changes may simulate residual tumor on routine microscopy, particularly when fibrosis is dense or associated with reactive atypia, requiring careful histology and immunohistochemistry (cytokeratins, p40, TTF-1, CD3/CD8/CD20/CD68) for accurate interpretation (9, 11).

These changes highlight the limits of chemotherapy-based criteria, which focus only on residual tumor percentage. This discrepancy has prompted the development of immune-adapted pathological frameworks, such as immune-related Pathologic Response Criteria (irPRC), which incorporate immune infiltration, fibrosis maturation, and spatial regression architecture into response evaluation (11, 13) (**Table 1**). At present, not fully standardized or universally accepted immune-adapted pathological criteria exist, and this lack of consensus contributes to variability in interpretation across institutions and among pathologists, particularly when evaluating complex immune-mediated regression patterns.

**Table 1:** Overview of potential immune-adapted histopathologic features of the tumor bed after neoadjuvant therapy.

Feature	Definition	Typical pattern (IO vs CT)	Common pitfalls	IHC/support	Suggested quantification	Supporting references
Residual viable tumor (RVT, %)	% of viable tumor cells within the total tumor bed	CT: reduced cellularity + necrosis; IO: rare scattered foci	Reactive cells mistaken for tumor; undersampling	Pan-cytokeratins, TTF-1/p40	Mapping of tumor bed + percentage estimate; AI-assisted segmentation	(9–11, 23)
Regression fibrosis	Dense collagen ± myofibroblasts	IO: extensive “mature” fibrosis; CT: fibrosis with necrosis	Fibrosis misinterpreted as residual tumor	CK-negative in fibrotic areas	Semi-quantitative scoring; stroma/tumor ratio with AI	(9, 11, 23)
Lymphocytic infiltrate (TILs)	Density of intratumoral and peritumoral lymphocytes	IO: prominent, organized	Overestimation of response if RVT present	CD3/CD8/CD20	Automated density (cells/mm²)	(11, 23, 24)
Tertiary lymphoid structures (TLS)	Organized aggregates with germinal centers	IO: frequent, mature	Misdiagnosis with reactive nodules	CD21/CD23, BCL6	TLS count/slide; maturation stage	(11, 24)
Granulomas/foamy macrophages	Histiocytes, giant cells, granulomas	IO: frequent, with lipid debris	Granulomas as tumor mimickers	CD68	Area % and granuloma count	(5, 9, 11, 23)
Necrosis/apoptotic debris	Necrotic foci, apoptotic debris	CT: extensive coagulative necrosis; IO: immune-mediated debris	Debris interpreted as RVT	—	% necrotic area; necrosis index	(9, 11, 23)
Cholesterol clefts	Linear slit-like spaces with foreign-body reaction	IO: clearing indicators	Misinterpreted as artifact	—	Count/area %	(11, 23)
Nodal regression	Fibrosis, pigmented histiocytes, sinus histiocytosis in nodes	IO: frequent	Loss of micrometastases in sampling	CK stains on fibrotic nodes	Dedicated nodal checklist	(17, 26)

Abbreviations: RVT; Residual Viable Tumor, CT; Chemotherapy, IO; Immunotherapy, TILs; Tumor-Infiltrating Lymphocytes, TLS; Tertiary Lymphoid Structures, IHC; Immunohistochemistry, CK; Cytokeratin.

Molecular profiling supports morphology in predicting immunotherapy response (5, 18). Tumor mutational burden (TMB) is a validated biomarker, with higher TMB linked to increased neoantigen load and enhanced immune recognition (1, 7). Other favorable signatures include Interferon Gamma, granzyme B, perforin, and checkpoint-related genes, reflecting active cytotoxic responses (5, 6). In contrast, mutations in oxidative stress pathways such as KEAP1–NFE2L2 are consistently associated with resistance and poor outcomes (12, 13). Moreover, an alternative immune escape mechanisms in ALK-positive NSCLC tumors has been explored, highlighting significant functional impairment of tumor-infiltrating CD8+ T cells despite low or absent PD-L1 expression on tumor cells (8). This finding highlights the existence of immunological resistance mechanisms beyond the canonical PD-1/PD-L1 pathway. Such mechanisms include intrinsic T-cell exhaustion, defective antigen presentation pathways, and alterations in immune co-stimulatory signals (6, 17, 26). Understanding these alternative resistance pathways through detailed molecular and immunologic profiling can inform rational design of combination therapies aimed at overcoming immunotherapy resistance in specific molecularly defined NSCLC subsets (5, 12, 18). From a clinical perspective, however, the only biomarker consistently validated across perioperative trials remains PD-L1, with lower response rates observed in PD-L1–negative tumors. Thus, in routine practice PD-L1 testing is most relevant on pre-operative biopsies, while comprehensive molecular or immunologic signatures are better explored on surgical specimens within research settings. Future strategies will likely rely on novel therapeutic combinations to broaden benefit in PD-L1–low/negative disease (32).

The integration of histopathological, molecular, and immunological evaluations is helpful for accurately predicting and interpreting therapeutic responses to immunotherapy in NSCLC (9, 10, 15). Pathologists play a central role in this integrated approach, maximizing advanced techniques, including immunohistochemistry, multiplex immunofluorescence, spatial transcriptomics, and next-generation sequencing, to comprehensively characterize tumor microenvironments and identify predictive biomarkers (5, 17, 18). Such comprehensive assessments not only enhance therapeutic response prediction but also provide critical insights into resistance mechanisms, guiding research and clinical trials aimed at further improving outcomes in NSCLC patients treated with immunotherapy (6, 7, 13).

## 5 Innovations and advances: digital pathology and AI integration

As mentioned above, the growing complexity of evaluating tumor regression following neoadjuvant therapies, particularly immunotherapy, has accelerated the integration of digital pathology and AI into routine diagnostic practice (5, 17, 24). These technologies are increasingly recognized as transformative tools in modern pathological anatomy, capable of enhancing diagnostic accuracy, minimizing interobserver variability, and improving standardization, especially in clinical trials and large-scale multicenter studies (9, 15, 18).

Histologic evaluation of post-immunotherapy specimens is challenging. Therapy-induced inflammation, necrosis, foamy macrophages, and remodeling of the tumor bed frequently overlap morphologically with viable tumor. Manual assessment can be prone to variability (9, 11, 24). Neverthless, recent studies have demonstrated that systematic protocols improve interobserver agreement and ensure acceptable reproducibility, important challenges remain (33). AI-based tools may provide reproducible quantification of viable tumor, necrosis, fibrosis, and immune infiltration. For example, AI algorithms can reliably detect small clusters of viable tumor cells embedded within dense fibrosis, areas that may be overlooked on manual review, especially in post-immunotherapy specimens where reactive stromal changes predominate (4, 34, 35). Unlike manual review, AI evaluates whole-slide images, reducing sampling errors and capturing focal tumor in fibrotic or inflamed areas. In addition, AI-based quantitative assessment of lymphocytic infiltrates and tertiary lymphoid structures provides far greater reproducibility than visual estimation. It can integrate multiple features simultaneously, providing a multidimensional assessment. Deep learning enables a truly multidimensional assessment by integrating patch-level morphology with aggregated pathomics signatures and clinical variables, improving prediction of MPR through a unified multimodal nomogram (4, 17). Beyond quantification, AI can detect spatial immune–tumor interactions, TLS density, or stromal activation, offering prognostic value and generating new research hypotheses (4, 19). Integrated into pathology platforms, AI highlights regions of interest and provides preliminary metrics, supporting but not replacing the pathologist, and enhancing efficiency and reproducibility (17, 24). For trials, AI offers standardized assessment across centers and facilitates integration with molecular and radiologic data, enabling predictive models that combine morphology, genomics, and immune dynamics (10, 19) (**Table 2**). Such tools are especially valuable given the current absence of unified immune-adapted pathological standards, which otherwise results in significant interobserver and inter-institutional variability in response interpretation. The implementation of AI in pathology requires rigorous validation, regulation, and quality assurance (5, 15, 17). However, despite their potential, digital pathology and AI solutions are still emerging technologies whose adoption requires substantial infrastructural investment, dedicated technical expertise, and external validation across different laboratories and scanning platforms. Most current evidence supporting these approaches comes from early-phase or retrospective studies, and prospective multicenter validation will be essential to confirm their reproducibility and clinical utility (9, 18, 24). Transparency in algorithm development, as well as interpretability of decision-making, remains a key ethical and scientific requirement (4, 10, 19).

**Table 2:** Example of multimodal integration matrix.

Archetype	Histology (pCR/MPR/RVT)	ctDNA (pre → post-neo → post-op)	Peripheral TCR (clonality/expansion)	Imaging (RECIST)	Integrated interpretation	Suggested clinical implications
A — “Immune complete responder”	pCR or RVT ≤10% with immune features (TLS, foamy macrophages)	High → not detectable	Significant clonal expansion	PR or SD (pseudoprogression)	Robust immune-mediated response	Standard surveillance; adjuvant optional/individualized
B — “Partial responder with MRD”	Borderline MPR (10–20%) or micro-RVT	High → decreased but persistent low-level	Modest expansion	PR	Possible MRD beyond sampling	Consider intensified adjuvant/clinical trial; close ctDNA follow-up
C — “Non-responder”	RVT >20%, poor immune features	High → persistent/increasing	No expansion	SD/PD	Biological treatment failure	Adjuvant switch/alternative therapy; serial ctDNA monitoring

pCR, Pathologic Complete Response; MPR, Major Pathologic Response; RVT, Residual Viable Tumor; ctDNA, Circulating Tumor DNA; TCR, T-cell Receptor; RECIST, Response Evaluation Criteria in Solid Tumors; TLS, Tertiary Lymphoid Structures; MRD, Minimal Residual Disease; PR, Partial Response; SD, Stable Disease; PD, Progressive Disease.

## 6 Emerging biomarkers: circulating tumor DNA and peripheral immune dynamics

Circulating tumor DNA (ctDNA) is a sensitive, minimally invasive biomarker that captures real-time tumor burden and residual disease, complementing histology and imaging (26, 36). After neoadjuvant immunotherapy, ctDNA can detect minimal residual disease (MRD) even when histology shows only fibrosis or immune stroma (6, 23). The NADIM trial demonstrated that ctDNA clearance strongly predicts MPR and improved survival, while persistent ctDNA correlates with relapse despite favorable imaging or pathology (7, 14). Thus, ctDNA dynamics provide a valuable adjunct for prognostication and adjuvant therapy decisions (15). Similarly, monitoring the T-cell receptor repertoire in blood reflects systemic immune engagement, and expansion of tumor-specific clonotypes during therapy correlates with intratumoral activity and MPR (5, 37). Both ctDNA and T-cell receptor (TCR) monitoring are most informative when integrated with pathology and radiology. Concordant clearance supports robust response, whereas persistence may alert for occult disease and guide closer follow-up (18, 26). Composite models combining these biomarkers with histology and imaging promise a multidimensional approach to treatment assessment (7, 12).

## 7 Clinical/pathological integration and prognostication

**Figure 3:**
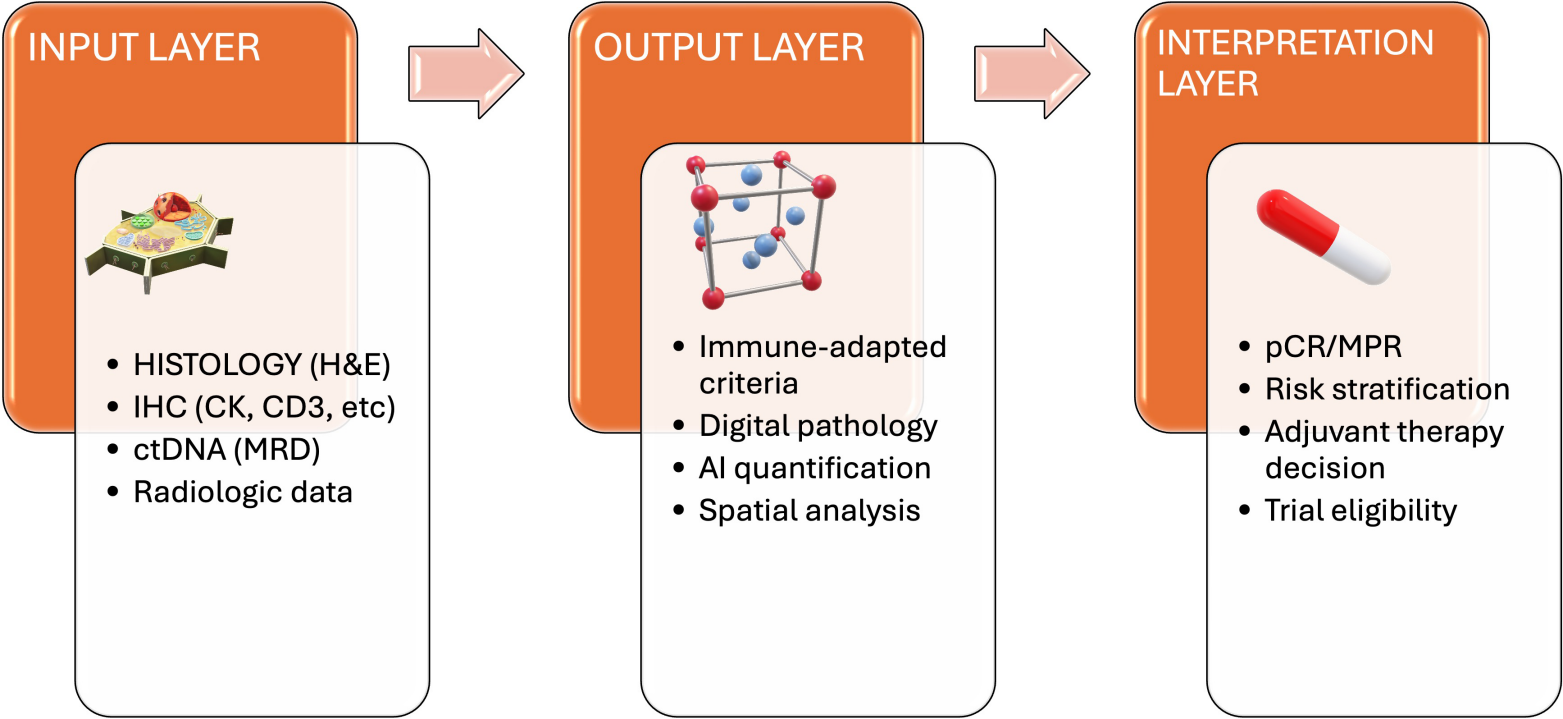
Conceptual framework for integrated pathological response assessment in NSCLC treated with neoadjuvant immunotherapy. The model begins with multimodal input layers, including traditional histopathology, liquid biopsy data, peripheral immune profiling, and radiologic information. These inputs are interpreted through immune-adapted criteria, AI-assisted digital pathology, and spatial analytic tools. The integrated output informs response classification, risk stratification, adjuvant therapy decisions, and clinical trial enrollment.

Equally important is refining biomarker accuracy, since current standards such as PD-L1 immunohistochemistry and TMB, although widely adopted, do not provide optimal performance (3, 13). To overcome these limitations, multi-dimensional biomarker panels have been proposed, combining morphological and molecular parameters with immune cell phenotyping, spatial distribution of infiltrates, transcriptional signatures, and liquid biopsy-derived information such as ctDNA or TCR clonality (5, 18, 37). The challenge lies not only in generating these heterogeneous datasets but in harmonizing and interpreting them in a clinically meaningful way. At present, the absence of harmonized protocols for integrating histologic, radiologic, and molecular data represents a significant barrier to broad adoption. In this context, machine learning models represent an essential integrative system, capable of processing high-dimensional data and identifying non-linear interactions between histopathological features, molecular alterations, and systemic immune markers that are often imperceptible to human evaluation (7, 18, 19). Importantly, such models can align pathological and molecular insights with radiologic and clinical parameters, thereby producing composite predictive algorithms that enhance biomarker precision and therapeutic stratification (4, 34). Pathologists remain central to this process, acting not only as curators of tissue morphology but also as gatekeepers of data quality, ensuring that digital pathology outputs, molecular profiles, and immune correlates are robust and clinically validated before they are integrated into machine learning pipelines (5, 7, 16). Ultimately, the convergence of pathology, molecular biology, and computational sciences through machine learning will enable the construction of predictive models that are reproducible, scalable, and applicable across institutions, facilitating a new generation of clinically relevant biomarkers (15, 18, 19) (**Figure 3**). Achieving this vision will require well-designed prospective multicenter studies, as most current data derive from retrospective cohorts with inherent limitations in standardization.

## 8 Conclusion

Neoadjuvant immunotherapy has reshaped NSCLC management and demands new ways of assessing response. Residual tumor percentage alone cannot capture the immune-driven changes in the tumor bed. Accordingly, conventional endpoints like MPR and pCR should be complemented by immune-adapted response criteria to improve accuracy and interpretability in the era of neoadjuvant immunotherapy. Pathologists must adopt integrated approach that combine immune features, digital pathology, and molecular biomarkers to standardize evaluation and guide therapy. Investing in consensus references and cross-disciplinary collaboration will be key to clinical translation. The tumor bed should now be seen as a dynamic record of tumor–host interactions. Refining its assessment will keep pathology central to precision medicine and improve patient outcomes. Harmonization of immune-adapted criteria remains a critical unmet need and will be essential to ensure reproducibility and comparability across clinical trials and diagnostic institutions. Future progress will depend on prospective, multicenter validation to establish reproducible and widely applicable standards.

RVT, Residual Viable Tumor; CT, Chemotherapy; IO, Immunotherapy; TILs, Tumor-Infiltrating Lymphocytes; TLS, Tertiary Lymphoid Structures; IHC, Immunohistochemistry; CK, Cytokeratin.
